# Factors Influencing Consumption of Edible Insects for Chinese Consumers

**DOI:** 10.3390/insects11010010

**Published:** 2019-12-20

**Authors:** Ai-Jun Liu, Jie Li, Miguel I. Gómez

**Affiliations:** 1 China Center for Food Security Studies, Nanjing Agricultural University, College of Economics & Management, Nanjing Agricultural University, Nanjing 210014, China; 2 Charles H. Dyson School of Applied Economics and Management, Cornell University, Ithaca, NY 14853, USA; jl2522@cornell.edu (J.L.); mig7@cornell.edu (M.I.G.)

**Keywords:** edible insect, consumer behavior, insect food, meat substitute, protein source, entomophagy

## Abstract

Edible insects are often considered a healthier and more sustainable meat substitute and protein source. Many studies have examined factors affecting the consumption behavior towards edible insects among Western consumers. However, little is known about factors influencing consumer behavior towards edible insects in Asian countries even though Asians have a long history of consuming insects. In this study, we surveyed 614 Chinese consumers from Beijing and Nanjing to examine the factors influencing their consumption and purchase behavior of edible insects. We find that insect phobia, feelings of disgust, knowledge level, and social demographic factors such as age, household size, household income and region (Northern or Southern China) are the main factors influencing purchase decisions. In addition, the results indicate that the perceived positive attributes associated with edible insects, the preferences of children in the household, as well as age and knowledge level have positive impacts on consumption frequency. On the other hand, concerns of food safety and the shape of the insects have negative impacts on consumption frequency. Finally, the results suggest that educating consumers to increase knowledge of edible insects increases their probability to purchase insect foods.

## 1. Introduction

With the world population expecting to grow to 9 billion by 2050, doubled agricultural production is needed to feed the people. Income growth in low- and middle-income countries is also accelerating the dietary transition towards higher meat consumption [1]. Increased meat demand, along with limited land area available for agriculture, may pose a challenge to the meat industry worldwide [2]. Conventional sources of meat protein may not be able to meet the needs of a growing global human population, and alternative sources such as insects are desirable [3]. The estimated value of the edible insect market was about 33 million dollars in 2015 in the US, Belgium, France, UK, Netherlands, China, Thailand, Vietnam, Brazil, and Mexico [3]. The market will likely grow to 522 million dollars by 2023, primarily driven by the growing consumer awareness and acceptance of edible insects in a global context [1,4].

As insects provide food at lower environmental cost (e.g., few greenhouse gases emissions, less feed, and much less farmland required), the Food and Agriculture Organization (FAO) and the United Nations (UN) have been promoting the consumption of edible insects among their member countries over the past several years [1]. Edible insects are also rich in proteins, vitamins, fats, and minerals. Moreover, rearing insects generally requires less technology input and less capital investment, which could potentially improve living conditions for poorer populations [5]. Rearing and consuming insects appear to be more desirable for developing countries. Particularly for a country like China where traditional agriculture production faces many challenges to feed 1.4 billion people due to limited farmland and resources. Accelerated urbanization further worsens the situation by taking over high-quality farmland and by speeding the rural-urban labor migration [6].

China has a long history of consuming insects (often referred to as entomophagy). Many insect species (e.g., silkworms, locusts, ants, honeybees and their products) are commonly consumed in different regions of China, and the level of acceptance for edible insects is relatively high in this country [7]. The Chinese Ministry of Health has recently promoted silkworm pupae as a new protein source to Chinese consumers, which has generated substantial research interest in this field [8]. The edible insect industry seems to have a complete and well-established supply chain and trade channels [9]. While many studies have been done focusing on consumers in Europe and the US [3,10,11,12], little is known about factors influencing Chinese consumer purchase and consumption decisions towards edible insects. To fill this gap, we surveyed 614 Chinese consumers from Beijing (Northern China) and Nanjing (Southern China) to examine the factors influencing their purchase and consumption decisions towards edible insects.

We conducted three analyses in this study: (1) we examined factors influencing consumers’ purchase decisions of edible insects, (2) we investigated factors influencing consumers’ consumption frequency towards edible insects, and (3) we studied whether educational information about edible insects influences consumer purchase decisions. The survey results suggest that consumers with a higher level of knowledge, a lower level of insect-phobia, fewer feelings of disgust, a larger household size, higher income, and living in the southern part of China are more likely to purchase edible insects. Additionally, higher knowledge level, more positive perception of attributes associated with insects, and older people tend to consume insects more often. In contrast, concerns about food safety and insect shape are likely to result in lower consumption frequency. We also find that providing educational knowledge of edible insects to consumers increases their purchase probability.

This research contributes to the current literature in two areas. First, previous consumer studies about edible insects have largely focused on developed countries. To our knowledge, this is the first quantitative study that collected data from the northern and southern parts of China to examine consumer attitudes and behaviors toward insects. This is particularly important, given that a sustainable and healthy meat substitute is desirable for developing countries. Second, previous literature has mostly examined factors influencing consumers’ attitudes toward edible insects. We extend the current literature by examining how to increase consumers’ adoption of this more sustainable protein source. This study has implications for policymakers and private practitioners interested in increasing consumers’ adoption of sustainable and healthier meat substitutes.

## 2. Materials and Methods

### 2.1. Survey Instruments

We designed a survey questionnaire to collect information regarding factors that influence consumer purchase decisions and consumption frequency towards edible insects. The questionnaire was finalized after a pilot study with consumers and repeated discussions with insect researchers. The questionnaire was pretested and then administered through face-to-face interviews. The respondents were informed that participation was voluntary and that they could withdraw at any time. The survey was conducted in metropolitan shopping areas in Beijing (the capital of China located in the northern part) and Nanjing (the capital of Jiangsu Province located in the southern part) in 2012.

The survey includes four parts: (1) consumer’s perception of insect food, perceived beneficial attributes of eating insects and reasons why a consumer likes or dislikes insect food; (2) importance ratings of purchase criteria (function, safety, insect shape, flavor, and brand of edible insects) for edible insects; (3) respondents’ purchase behavior when provided with educational information about edible insects; and (4) an exit survey eliciting demographic information including age, gender, education, experience of edible insects, income levels, and birthplace.

### 2.2. Empirical Model

In this study, we conducted three analyses to examine factors influencing Chinese consumer purchase and consumption behavior. First, we employed a logit model to examine the factors influencing consumer purchase decisions of edible insects. We then used an ordered logit model to investigate the main determinants influencing consumers’ consumption frequency. We next specified a logit model analysis to examine whether and to what extent educational information influences purchase decision. We discuss these empirical models sequentially below.

#### 2.2.1. Logit Model of Factors Influencing Purchase Decisions

The empirical model could be expressed as

1. Purchase decision = *F*_1_ [Insect phobia, Feelings of disgust, Knowledge level, Demographics (Age, Birthplace, Household income, Household size, City dummy)]

Regarding the dependent variable, we asked respondents whether they have purchased edible insects before. The dependent variable, denoted as Y binary, equals one if respondents purchased edible insects before and zero otherwise.

Regarding the explanatory variables, in the survey, we asked respondents about the main reason that affects their purchase decisions. We generated a dummy variable *Insectphobia* that equals one if participants choose insect phobia as the primary reason for not buying edible insects and zero otherwise. We also generated a variable labeled as *Disgust* that equals one if participants choose the feelings of disgust as the primary reason for not buying edible insects and zero otherwise.

Additionally, previous literature suggests consumer knowledge of edible insects had significant impacts on their attitudes toward insect food [10]. We therefore included the variable *Knowledgelevel* to measure participants’ knowledge level about edible insects on a scale of one to four, with one indicating not knowing about edible insects and four indicating high knowledge about edible insects.

Participants’ social demographics were also included in our model as control variables such as education, age, and gender [13,14,15,16,17]. We also included respondents’ birthplace, income level, and household size to capture characteristics unique to Chinese consumers. We created a variable *Birthplace* to control the effect of cultural influence on food preferences. The *Birthplace* variable equals one if a respondent was born in Northern China and zero otherwise. Participants’ age group is denoted as *Age*. This variable has six levels, with level one being the lowest age level (18–24) and level six being the highest level (65+). The annual household income level, denoted as *Householdincome*, was measured on a scale from one to nine, with one being the lowest level (less than 30,000 RMB/4762 dollars) and nine being the highest level (more than 210,000 RMB/33,333 US dollars). The *Householdsize* variable measures the number of people living with the respondent. We also included a *Citydummy* variable to control the city-specific effect. The *Citydummy* variable equals one if the survey was collected from Nanjing, and equals zero if collected from Beijing. Northern and southern China consumers tend to have different food preferences [17].

#### 2.2.2. Ordered Logit Model of Consumption Frequency

The empirical model for the ordered logit regression is expressed as:

2. Consumption Frequency = F_2_ [Perceived number of beneficial attributes, Importance rating of purchase criteria, Kids preference level, Knowledge level and Demographics (Age, Income, Household size, and City dummy)].

Regarding the dependent variable, we focused only on the participants who had purchased and consumed edible insects before in order to examine the determinants influencing their consumption frequency towards edible insects. We asked the respondents to identify their consumption frequency over the past year, denoted as *Y_CF*. This variable includes three levels and equals one if participants have only consumed a few times, equals two if participants have consumed on a regular basis but not quite often, and equals three if participants have consumed the edible insects on a regular basis.

Regarding explanatory variables, respondents were asked about the perceived positive attributes associated with edible insects from a list of 10 attributes. A variable *Attributes* was generated for each respondent defined as the number of attributes they selected in the survey (These attributes include high nutrition, high protein, low fat, low cholesterol, fresh food, good taste, strengthen the immune system, treatment for diseases, anti-aging, and traditional food). Several variables, denoted as *Function*, *Safety*, *Insectshape*, *Flavor*, and *Brand*, were generated to measure the importance ratings of respondents on purchase criteria (i.e., function, safety, insects shape, flavor, and brand of edible insects). The rating ranges from one to six, with one being not important and six being very important. The preference level of the participants’ kids (labeled as *Preference_kid*) was measured from one to four, with one being not liking it at all and four indicating liking it very much. Age, household size, income, and city dummy were also controlled in this model.

#### 2.2.3. Simple Logit Model to Estimate the Information Treatment on Choices

After participants completed the first three parts of the survey questionnaire, they were provided some educational information about edible insects. Then the participants were asked again about their willingness to purchase edible insects in the future. The information we provided to consumers about edible insects was written in Chinese, and we translated them into English, which reads as: “In Compendium of Materia Medica, there are 40 or 50 species of insects commonly used in Chinese medicine. In Compendium of Materia Medica, Huang Di Nei Jing, and other traditional Chinese medicine classics, anti-aging insects are frequently recorded. For example, Compendium of Materia Medica: ‘Ant power is the greatest, can lift heavy things, improve energy, and moisturize skins.’ In Compendium of Materia Medica, the male silkworm moth is considered to have the magical effect of strengthening the liver and kidney alongside possessing anti-aging effects. Some studies have shown that there are 18 kinds of amino acids and eight kinds of essential amino acids in the body of *Tenebrio molitor*, which accounts for 40% of the total amino acid content, and the proportion of them is close to the amino acid requirement of the human body. Moreover, *Tenebrio molitor* also contains a variety of minerals, trace elements, and vitamins. They also contain a high content of riboflavin and vitamin E. This is important, as vitamin E is an indispensable nutrient source and possess medicinal value, which can protect lipids from peroxidation.”.

The dependent variable *Y_Choice* is coded as one if participants responded that they have purchased (or are willing to purchase in the future) edible insects, zero otherwise. A dummy variable (labeled as *Information*) to capture the information treatment is also generated. This variable has two values: one represents consumer choices after the information treatment, and zero represents consumers’ choices before the information treatment. As the main purpose of this analysis is to examine how educational information influences consumer purchase decision before and after, we, therefore, conducted a simple logit regression. The empirical model is expressed as

3. Purchase decision = F_3_ [Information treatment].

### 2.3. Data

A total of 614 usable questionnaires were collected. The data preparation process included questionnaire checking, editing, coding, transcribing, cleaning the data, and selecting a data analysis strategy. We used STATA 16.0 software, a statistical software package created by StataCorp, for the analyses [18].

Table 1 presents the descriptive statistics for the logit regression model to estimate consumer purchase decisions. The *Y_binary* has a mean of 0.391, indicating about 240 of the total respondents had purchased edible insect food before. The mean of *Insect_phobia* is 0.588, indicating 58% of the participants chose insect-phobia as the main reason for not buying insects. The mean value of *Disgust* is 0.21, indicating that 21% of respondents chose the feeling of disgust as the primary reason for not consuming insects. The mean value of *Birthplace* suggests that 44.95% of respondents were born in the Southern part of China. The mean value of *Citydummy* shows that 65% of the surveys were collected from Nanjing. The average knowledge level of the edible insect is 1.344, indicating that most respondents reported having very limited knowledge. For *Age* and *Householdincome*, the mean values are 2.733 and 2.627, respectively, which indicate that most respondents are young and in lower-income groups. The mean value of *Householdsize* is 3.845 as the result of the one-child policy and extended family culture (where several generations live in the same household). 

Table 2 presents the descriptive statistics for the ordered logit model to estimate consumers’ insect consumption frequency. The dependent variable *Y_CF* has three levels with a mean of 1.5. The average number of perceived beneficial attributes associated with edible insects is 1.956 (close to 2), indicating respondents on average perceive two beneficial attributes associated with consuming edible insects. For the importance ratings of purchase criteria for edible insects, all the mean values are higher than three, indicating that on average respondents think that these criteria are important. *Safety* has the highest value (5.49), suggesting that food safety is the most important purchase criteria perceived by respondents. The mean of *Preference_kid* is 1.632, indicating on average, kids have a lower preference level for edible insects. Participants’ knowledge level of edible insects is 1.539 on average, and 3% of participants have zero knowledge, 48% of the sample has limited knowledge, 47% of the sample has some knowledge, and only 2% know edible insects quite well. The mean value of *Age* is 2.985, indicating that most respondents are less than 35 years old. Regarding the *Householdincome* variable (2.877 mean value), most of the sample has an income of less than CNY 70,000. The average household size in this sample is 4.004. In our sample, 74% of respondents are from Nanjing, in the southern part of China.

## 3. Results

We present results for the models with *Y_binary* (regression 1) and *Y_CF* (regression 2) as dependent variables in Table 3 and Table 4, respectively. We next discuss the simple logit regression (regression 3) estimating the impacts of educational information on consumers’ purchasing decisions.

### 3.1. Regressing 1: Logit Model of Factors Influencing Purchase Decisions

Columns 2 and 3 in Table 3 present the estimated coefficients and marginal effects of the explanatory variables for the model with *Y_binary* as the dependent variable. Regarding the reasons hindering consumer purchase decisions, the results show that the coefficient of *Insect_phobia* and *Disgust* are positive and significant, suggesting that insect phobia and feelings of disgust for edible insects are the main factors hindering the purchase of edible insects. The marginal effects indicate that the probability of purchasing edible insects is 12% and 13% lower when respondents choose insect phobia and feelings of disgust, respectively, as the main reasons for not buying edible insects.

The estimation results in Table 3 show that *Knowledgelevel* has a significant negative effect on edible insect purchase, and a one-level increase in consumers’ knowledge level increases the possibility of purchasing edible insects by 20%. Regarding the social-demographic factors, the results show that the coefficient of *Age*, *Householdincome*, and *Householdsize* are positive and statistically significant, indicating when respondents are older, have higher income, and have a larger household size, they are more likely to purchase edible insects. The *Citydummy* variable is also positive and significant, indicating that respondents in Nanjing are more likely to purchase edible insects relative to the respondents in Beijing, reflecting the cultural influence on food preferences and choices (People in the Northern China have a wheat-based diet, including noodles, breads, dumplings, and saltier foods (to preserve); people in the Southern china have rice-based diet. They prefer sweeter food, more sugar, and consume more seafood. They are famous for “Eat anything with four legs but a table, everything that flies but a kite or plane”. (https://www.barrypopik.com/index.php/new_york_city/entry/they_eat_any thing_with_legs_except_a_table_and_anything_with_wings_except_a, accessed by 2 December 2019). The results show that Birthplace has no impact on purchase decision.

### 3.2. Regression 2: Ordered Logistic Regression

In this regression, we focused only on the 204 consumers who had previously purchased and consumed edible insects to examine the factors influencing their consumption frequency. The dependent variable *Y_CF* takes one of three values: low, medium, and high consumption frequency. The LR Chi Square indicates that the null hypothesis of all coefficients being equal to zero is rejected at the 1% level of significance (Table 4).

The coefficient of *Attribute* is positive and statistically significant, indicating that the perceived number of beneficial attributes of edible insects has impacts on consumption frequency. With a one-unit increase in the number of perceived beneficial attributes, respondents are much more likely to have a higher degree of consumption of edible insects than a lower one. The odds ratio is 1.619, indicating that a one-unit increase in the perceived number of beneficial attributes associated with edible insects increases the odds of being in the high consumption frequency category by 48.2%. For a list of importance ratings for the purchase criteria of edible insects, the coefficients of *Safety* and *Insectshape* are both negative and statistically significant. The results indicate that the higher importance rating of food safety leads to a lower consumption frequency. The odds ratio is 0.640, which indicates that a one-unit increase in safety concerns decreases the odds of being in the higher consumption frequency category by 44.6%. Similarly, the higher importance rating of the insect shape leads to a lower consumption frequency. The odds ratio is 0.839 indicates that a one-unit increase in concerns about insect shape decreases the odds of being in the higher consumption frequency category by 17.5%. However, the coefficients for *Function*, *Brand*, and *Flavor* are not significant.

The coefficient for *Preference_kid* is positive and significant, indicating that a higher preference level of the kids in the household for edible insects leads to a higher consumption level. This makes sense, as in China, the family’s meal choices heavily rely on the kids’ preferences, probably due to the one-child policy and parenting cultures. The *Knowledgelevel* variable is significant and positive, and the odds ratio is 1.556, which indicates that a one-unit increase in consumers’ knowledge level increases the odds of being in the higher consumption frequency category by 44.2%. This result suggests that limited knowledge of edible insects seems to be the factor hindering consumers from choosing insects for food.

For demographic variables, the coefficient of *Age* is positive and statistically significant, indicating that older respondents tend to consume more edible insects. This might be because edible insects were used as the main food source during the Great China Famine. As a result, older generations are more familiar with edible insects and therefore are likely to have a higher consumption frequency. The results for *Householdincome*, *Householdsize*, and the *Citydummy* are not significant under this model specification.

### 3.3. Regression 3: Logit Regression to Estimate the Impact of Information

We specified a simple logit regression to examine the impact of educational information on consumer purchase decisions. The results indicate that the educational information has a positive and significant impact on their choices (SD = 0.114, Z = 6.65, *p* < 0.0001). The marginal effect is 0.188 (SD = 0.276, Z = 6.81, *p* < 0.001), indicating that after providing participants the educational information, their probability of making the purchase decision increases by 18.8%. Educating participants with knowledge of edible insects may reduce the fear of insects and increases their purchase probability. This result seems consistent with Regressions 1 and 2 that consumers’ knowledge about edible insects is the main factor driving their purchase and consumption decisions.

## 4. Discussion and Conclusions

Insects play an important role in addressing the ongoing global nutritional challenges. This is because insects contain sufficient levels of protein, fats, and micronutrients, which could substantially improve global health and food security through direct consumption and indirect use in animal feeds [9]. Apart from Europe and North America, approximately 1500–2000 species of insects and other invertebrates are consumed by 3000 ethnic groups across 113 countries in Asia, Australia, and Central and South America [13]. It is estimated that at least two million people, mostly living in the tropical and subtropical countries, include edible insects (about 1900 insect species) in their diet on a regular basis [14]. Insects can be eaten raw, fried, boiled, roasted, or ground at various life stages. A recent study by [15] shows that around 2111 known edible insect species are currently consumed. These insect species tend to be abundant and are easier to capture (e.g., they are generally wingless and slow-moving) [16,17]. Although the insect industry has been growing quickly over the past five years, the industry still faces many challenges that require collaborative actions from all stakeholders [19,20,21,22,23].

China has a long history of consuming edible insects. In Ancient China, Lu You of the Song Dynasty and Kuang Lu of the Ming Dynasty introduced consuming of ants by people in Guangxi [18]. These traditions of eating insects continue today and are well inherited in many parts of China by different ethnic groups. For example, dishes made from ants are well known in some southern cities in China, such as “ants hugging eggs” and “ants climbing trees”, to name a few [18]. Insects are mostly raised and bred in rural China for human consumption, medicinal purposes, and feeding animals [18]. It is estimated that 178 insect species from 96 genera, 53 families, and 11 orders are commonly consumed today [19,24]. Eggs and adults are mostly processed and prepared for snacks, while larvae and pupae are mostly consumed as main courses in restaurants. Preparation of edible insects includes deep-frying, braising, stewing, stewing after frying, boiling, and roasting. There are 20–30 popular species consumed in restaurants year-round, including grasshoppers, silkworm pupae, wasps, bamboo insects, and stink bugs [20]. China also has a complete insect supply chain, including commercial farms as well as well-established trade channels [7].

Previous studies focusing on European consumers’ behavior toward edible insects are well documented. For example, the authors of [12,25] argue that European consumers, particularly younger generations, only exhibit a positive attitude to insect food if insects are mixed with regular meat products. They also find that consumers’ responses rely heavily on insect shape and appearance. In [26], the authors find that consumers have a lower preference for proteins made from insects (i.e., snacks made with locusts) relative to the ones made from other environmentally friendly protein sources such as lentils, seaweed, or hybrid meat. Another study explores young Italians’ attitudes toward edible insects using a focus group interview. The authors find that the form of the product appears to be a significant factor influencing participants’ willingness to eat [27].

More recently, a study reports that curiosity is the main reason for Italians to try edible insects, while feelings of disgust and negative opinions from family and friends about eating insects are the main barriers [17]. Germans tend to have a higher willingness to eat processed insect-based foods compared to unprocessed foods [21,22,28] suggests that repeated consumption of edible insects is mainly affected by the same factors influencing general food choices among Dutch consumers. A recent study researched consumer acceptance of edible insects in Belgium after insect-based food was introduced to the market. They found that most people are aware of such insect-based food products and they also found that to declare the products that contain insects is important for consumers [29].

On the other hand, limited scientific research is available focusing on Asian consumers. Some anecdotal evidence [23] suggests that women in Thailand consume more insects when they are pregnant, particularly bee nests, because they believe eating insects benefit the baby. In the case of South Korea, insect-based food has been a part of standard diets and the government continues encouraging the adoption of edible insects in Korean cuisine. However, younger generations appear to have a negative attitude toward eating unprocessed insects as food. Korean consumers are willing to choose the edible insect cookie if it is combined with other familiar ingredients such as wheat flour and milk [30,31,32].

Previous studies also show that Chinese consumers are more familiar with the idea of consuming insects relative to Western consumers and therefore Chinese consumers have higher acceptances of consuming edible insects [7,21,33,34,35]. However, even though China plays an important role in farming and consuming edible insects, reputable studies focusing on edible insect markets, consumers, and the supply chain in China are very limited [36,37,38,39]. To fill this gap, we collected data from 614 survey respondents from Beijing and Nanjing of China to study Chinese consumer purchase and consumption behaviors as well as examine the influence of educational information of edible insects on consumer choices.

Our results show that insect phobia and feelings of disgust are the main reasons hindering consumers from consuming edible insects. Knowledge level, age, household size, and household income also influence consumer purchase decisions about edible insects. Regarding factors influencing consumption frequency, we find that the perceived number of beneficial attributes associated with edible insects, knowledge level, and age have a positive impact on consumption frequency, and food safety concerns and insect shape have a negative impact on consumption frequency. Finally, the estimation results suggest that educating consumers with edible insect knowledge increases their purchase likelihood.

### 4.1. Implications

Our findings suggest that insect phobia and feelings of disgust are still the major factors hindering their purchase decisions. Reducing consumers’ insect phobia and feelings of disgust could have the potential to boost sales. This may require collaborative marketing actions from policymakers and private business (producers and marketers) to educate consumers about the benefits of edible insects. Increased consumer knowledge about edible insects would result in new customers who are willing to try this meat substitute, and it also increases the consumption frequency of repeated consumers.

Food safety has been a very serious issue facing the food industry in China. From gutter oil to melamine-tainted milk, and from fake meat to contaminated strawberries, these scandals caused great anxiety among Chinese consumers. Concerns about food safety of edible insects appear to be the main factor affecting consumers’ consumption frequency. Effective government policy to make sure the process and preparation of edible insects strictly follow the desired food safety guidelines are necessary.

It also appears that the shape of edible insects is another main factor hindering consumers’ consumption frequency. The industry could develop novel products that use insects as ingredients but avoid presenting the form of the insects. For example, a protein bar made from Crickets has been developed and widely marketed in the US market. Additionally, the industry could work on developing marketing strategies (e.g., better package) to attract curious consumers, which might be more effective than convincing consumers that eating bugs is healthy [33].

This study provides insights to producers and marketers about how to target consumer segments of edible insects. Promotional efforts should be heavily given to older consumers and households with kids, as the results above suggested. Furthermore, the higher income consumer segment may also be the appropriate targeted consumer segment. This is because edible insects as an innovative healthy protein source may be preferred among this group of consumers who have high disposable income and pay more attention to a healthy and balanced diet. It is very common for finer restaurants to serve edible insects as a unique dish targeting higher-income people.

### 4.2. Limitations and Future Research

While this study provides valuable information for policymakers and private practitioners, there are several limitations that should be addressed by future research. We conducted our survey from two cities in China, Beijing (the capital city of China located in Northern China) and Nanjing (the capital city of Jiangsu Province located in Southern China). Future research could broaden the scope of this study to collect a more diverse consumer sample. Future research could also investigate the influence of cultural differences on consumer purchase behavior of edible insects. Future studies could examine how educational information influences consumers’ consumption decisions when considering their cultural influence. Lastly, future research could extend this study using samples from other less developed Asian countries to examine consumer acceptance of this new lower cost protein source.

## Figures and Tables

**Table 1:** Descriptive statistics for logit model to estimate consumer purchase decisions.

Variable	Description	Mean	Standard Deviation	Min	Max
**Dependent Variables**					
*Y_binary*	Whether participant purchased edible insects or not (yes = 1, no = 0)	0.391	0.488	0	1
**Independent Variables**					
*Insect_phobia*	=1 if participants choose afraid of insects as the primary reason for not buying edible insects, =0 otherwise	0.588	0.493	0	1
*Disgust*	=1 if participants choose *Disgust* as the primary reason for not buying edible insects, =0 otherwise	0.205	0.404	0	1
*Knowledgelevel*	=1 if participants have zero knowledge of edible insects, =2 if participants have a little knowledge, =3 if participants have some knowledge, and =4 if participants have lots of knowledge	1.344	0.563	0	3
*Birthplace*	=1 if participants were born in the northern part of China, =0 otherwise	0.450	0.498	0	1
*Age*	The age group of the participant	2.733	1.489	1	6
*Householdincome*	The household annual income level of each participant	2.627	1.433	1	6
*Householdsize*	The number of people living with the participant	3.845	1.091	2	8
*Citydummy*	=1 if the survey was collected in Nanjing located in the southern part of China, =0 if the survey was collected in Beijing	0.653	0.476	0	1

**Table 2:** Descriptive statistics for ordered logit model.

Variable	Description	Mean	Standard Deviation	Min	Max
**Dependent Variables**					
*Y_CF*	=1 if participants have a low CF, =2 if participants have a medium CF, and =3 if participants have a high CF	1.500	0.608	1	3
**Independent Variables**					
*Attributes*	The number of benefits of eating edible insects that each participant identified	1.956	1.376	0	10
*Function*	The importance rating of each participant for the function factor when making purchase decisions	4.706	1.564	1	6
*Safety*	The importance rating of each participant for the safety factor when making purchase decisions	5.490	1.129	1	6
*Insectshape*	The importance rating of each participant for the shape of the insect factor when making purchase decisions	3.397	1.802	1	6
*Flavor*	The importance rating of each participant for the flavor factor when making purchase decisions	4.765	1.520	1	6
*Brand*	The importance rating of each participant for the brand factor when making purchase decisions	3.098	1.759	1	6
*Preference* _ *kid*	The preference level of participant’s kids for edible insects	1.632	0.841	1	4
*Knowledgelevel*	=0 if participants have zero knowledge of edible insects, =1 if participants have a little knowledge, =3 if participants have some knowledge, and =4 if participants have lots of knowledge	1.539	0.573	0	3
*Age*	The age group of the participant	2.985	1.423	1	6
*Householdincome*	The household income level of each participant	2.877	1.435	1	6
*Householdsize*	The number of people in the household	4.044	1.188	2	8
*Citydummy*	=1 if the survey was collected in Nanjing, =0 if the survey was collected in Beijing	0.740	0.440	0	1

**Table 3:** Logit model for the decision to buy or not buy.

Variable Name	*Y_binary (Purchase or Not)*	
	Coefficients	Marginal Effects
*Insect_phobia*	−0.506 **	−0.120 **
	(0.232)	(0.055)
*Disgust*	−0.598 **	−0.133 **
	(0.285)	(0.059)
*Knowledgelevel*	0.846 ***	0.199 ***
	(0.161)	(0.038)
*Birthplace*	0.446	0.105
	(0.273)	(0.064)
*Age*	0.121 *	0.027 *
	(0.062)	(0.014)
*Householdincome*	0.156 **	0.037 **
	(0.062)	(0.015)
*Householdsize*	0.225 ***	0.053 ***
	(0.082)	(0.019)
*Citydummy*	1.095 ***	0.242 ***
	(0.298)	(0.061)
Number of observations	614	-
Log likelihood	−372.061	-
LR Chi Square	77.58	-
Prob > Chi Square	0	-
Pseudo R^2^	0.0944	-

Notes: The number of observations in the model is 614. We evaluated the difference of the probability of 1 and 0 for the discrete variables while holding all other variables at their means. For continuous variables, we obtained the marginal effects by taking the derivatives of the variable while fixing all variables at the mean. We employed STATA for estimation. *, **, and *** denote coefficient estimates that are statistically significant at the 0.10, 0.05, and 0.01 level, respectively. Standard errors are presented in parentheses. Each variable is defined in Table 1.

**Table 4:** Ordered logistic regression results and marginal effects.

Variable Name	Three Consumption Levels				
	Coefficients	Odds Ratio	Low CF	Medium CF	High CF
			Marginal Effect	Marginal Effect	Marginal Effect
*Attributes*	0.482 ***	1.619 ***	−0.086 ***	0.064 ***	0.022 ***
	(0.129)	(0.210)	(0.021)	(0.017)	(0.007)
*Function*	0.025	1.025	−0.004	0.003	0.001
	(0.116)	(0.119)	(0.021)	(0.016)	(0.005)
*Safety*	−0.446 ***	0.640 ***	0.080 ***	−0.060 ***	−0.020 **
	(0.156)	(0.0998)	(0.026)	(0.020)	(0.008)
*Insectshape*	−0.175 *	0.839 *	0.031 *	−0.023 *	−0.008
	(0.106)	(0.0889)	(0.019)	(0.014)	(0.005)
*Flavor*	−0.067	0.935	0.012	−0.009	−0.003
	(0.121)	(0.113)	(0.022)	(0.016)	(0.006)
*Brand*	0.142	1.152	−0.025	0.0190	0.006
	(0.107)	(0.124)	(0.019)	(0.014)	(0.005)
*Preference_kid*	0.442 **	1.556 **	−0.079 **	0.059 **	0.020 *
	(0.197)	(0.307)	(0.034)	(0.025)	(0.010)
*Knowledgelevel*	0.805 ***	2.237 ***	−0.145 *	0.108 ***	0.037 **
	(0.301)	(0.674)	(0.515)	(0.039)	(0.016)
*Age*	0.287 **	1.332 **	−0.051 **	0.038 **	0.013 **
	(0.119)	(0.158)	(0.021)	(0.016)	(0.006)
*Householdincome*	0.067	1.069	−0.012	0.009	0.003
	(0.118)	(0.126)	(0.021)	(0.016)	(0.005)
*Householdsize*	−0.104	0.902	0.019	−0.014	−0.005
	(0.142)	(0.128)	(0.025)	(0.019)	(0.007)
*Citydummy*	0.162	1.176	−0.029	0.022	0.007
	(0.401)	(0.472)	(0.072)	(0.054)	(0.018)
Number of observations	204				
Log likelihood	−139.966				
LR Chi Square	70.72				
Prob > Chi Square	0				
Pseudo R^2^	0.2017				

Notes: The number of observations is 204 instead of 240 because some values were missing when the independent variable was constructed. Three levels of consumption: low consumption frequency (value = 1), medium consumption frequency (value = 2), high consumption frequency (value = 3). We evaluated the difference of the probability of 1 and 0 for the discrete variables while holding all other variables at their means. For the continuous variables, we obtained the marginal effects by taking the derivatives of the variable while fixing all variables at the mean. We employed STATA for estimation. *, **, and *** denote coefficient estimates that are statistically significant at the 0.10, 0.05, and 0.01 level, respectively. Standard errors are presented in parentheses. Each variable is defined in Table 2.
